# Cardiovascular magnetic resonance predicts all-cause mortality in pulmonary hypertension associated with heart failure with preserved ejection fraction

**DOI:** 10.1007/s10554-021-02279-z

**Published:** 2021-05-12

**Authors:** Pankaj Garg, Robert A. Lewis, Christopher S. Johns, Andrew J. Swift, David Capener, Smitha Rajaram, A. A. Roger Thompson, Robin Condliffe, Charlie A. Elliot, Athanasios Charalampopoulos, Abdul G. Hameed, Alexander Rothman, Jim M. Wild, David G. Kiely

**Affiliations:** 1 Department of Infection, Immunity and Cardiovascular Disease, University of Sheffield, Sheffield, S102JF England; 2 Sheffield Teaching Hospitals NHS Foundation Trust, London, England; 3 Sheffield Pulmonary Vascular Disease Unit, Royal Hallamshire Hospital, Sheffield, S10 2JF England

**Keywords:** Magnetic resonance imaging, Heart failure, Pulmonary hypertension, Prognosis, Right ventricular function

## Abstract

This study aimed to determine the prognostic value of cardiovascular magnetic resonance (CMR) in patients with heart failure with preserved ejection fraction and associated pulmonary hypertension (pulmonary hypertension-HFpEF). Patients with pulmonary hypertension-HFpEF were recruited from the ASPIRE registry and underwent right heart catheterisation (RHC) and CMR. On RHC, the inclusion criteria was a mean pulmonary artery pressure (MPAP) ≥ 25 mmHg and pulmonary arterial wedge pressure > 15 mmHg and, on CMR, a left atrial volume > 41 ml/m^2^ with left ventricular ejection fraction > 50%. Cox regression was performed to evaluate CMR against all-cause mortality. In this study, 116 patients with pulmonary hypertension-HFpEF were identified. Over a mean follow-up period of 3 ± 2 years, 61 patients with pulmonary hypertension-HFpEF died (53%). In univariate regression, 11 variables demonstrated association to mortality: indexed right ventricular (RV) volumes and stroke volume, right ventricular ejection fraction (RVEF), indexed RV mass, septal angle, pulmonary artery systolic/diastolic area and its relative area change. In multivariate regression, only three variables were independently associated with mortality: RVEF (HR 0.64, P < 0.001), indexed RV mass (HR 1.46, P < 0.001) and IV septal angle (HR 1.48, P < 0.001). Our CMR model had 0.76 area under the curve (P < 0.001) to predict mortality. This study confirms that pulmonary hypertension in patients with HFpEF is associated with a poor prognosis and we observe that CMR can risk stratify these patients and predict all-cause mortality. When patients with HFpEF develop pulmonary hypertension, CMR measures that reflect right ventricular afterload and function predict all-cause mortality.

**Supplementary Information:**

The online version contains supplementary material available at 10.1007/s10554-021-02279-z.

## Introduction

Heart failure with preserved ejection fraction (HFpEF) now constitutes approximately half of all heart failure (HF) diagnoses [1]. It is estimated that approximately 50% of patients with HFpEF develop pulmonary hypertension [2, 3]. The development of pulmonary hypertension in HFpEF is mainly due to raised left ventricular filling pressures, which causes a sustained backward hemodynamic transmission to the pulmonary vascular bed. In patients with HFpEF, the development of pulmonary hypertension predicts a worse prognosis [2, 4].

Cardiovascular magnetic resonance (CMR) is the imaging reference standard for volumetric assessment and is emerging as one of the key non-invasive imaging methods to diagnose, and temporally monitor patients with pulmonary hypertension [5, 6] and phenotype patients with HFpEF [7]. The guidelines for the diagnosis and treatment of pulmonary hypertension highlight the potential of CMR in the assessment of disease severity and follow-up [8–10].

Cardiac MR provides added value to the clinical assessment in the prognostic evaluation of patients with pulmonary arterial hypertension [11] and allows for improved risk stratification of patients with PAH when used in conjunction with the REVEAL 2.0 and the modified French Pulmonary Hypertension Registry risk score [12]. In HFpEF, CMR can also sub-phenotype myocardial diseases that lead to the development of myocardial stiffness [13], including cardiac amyloidosis, hypertensive cardiomyopathy, hypertrophic cardiomyopathy and cardiac sarcoidosis.

Currently, there are limited outcome data in advanced stages of HFpEF using CMR, especially in patients with pulmonary hypertension-HFpEF. In addition, it remains unknown if CMR can be used in this patient population to risk stratify patients and predict prognosis [9].

The aim of this study was to investigate if non-contrast enhanced cine and flow CMR could risk stratify patients with pulmonary hypertension-HFpEF and inform prognosis.

## Methods

This study was approved by the National Research Ethics Service (16/YH/0352) in the UK. The study complied with the Declaration of Helsinki. Patients were identified from the ASPIRE registry [14] and underwent a detailed assessment including blood, lung function, exercise testing, multimodality imaging and right heart catheterisation as previously described [14, 15]. Consecutive patients with suspected pulmonary hypertension who underwent CMR from April 2012 to April 2017 were assessed for inclusion in the study. All patients were recruited at Sheffield Teaching Hospitals NHS Foundation Trust. Inclusion criteria included age ≥ 18 years and a confirmed diagnosis of pulmonary hypertension-HFpEF. A diagnosis of pulmonary hypertension-HFpEF required a mean pulmonary arterial pressure (mPAP) ≥ 25 mmHg with a pulmonary artery wedge pressure (PCWP) > 15 mmHg at right heart catheterisation (RHC) and a left ventricular ejection fraction > 50% with a left atrial volume index > 41 ml/m^2^ on CMR [16]. Right heart catheterisation was performed using a balloon-tipped 7.5Fr thermodilution catheter (Becton-Dickinson, Franklin Lakes, New Jersey). The mean pulmonary arterial pressure and pulmonary artery wedge pressure were recorded using standard techniques described previously [17]. Patients with other forms of pulmonary hypertension were excluded.

### CMR acquisition

CMR was performed on a GE HDx 1.5-T system (GE Healthcare, Milwaukee, Wisconsin) using an 8-channel cardiac coil.

The protocol included four-chamber (4Ch) and short-axis (SA) cine images, acquired using a retrospectively cardiac gated multi-slice steady-state free precession (SSFP) sequence. We acquired a stack of axial images in the short axis (SA) plane, with a slice thickness of 10 mm with no inter-slice gap or 8 mm with a 2 mm inter-slice gap, from the base to the apex of both ventricles. Time-resolved images of the pulmonary artery were performed using a retrospectively cardiac gated SSFP sequence with a single slice of 10 mm taken perpendicular to the long-axis of the pulmonary artery. The SSFP sequence parameters were: TR 2.8 ms, TE 1.0 ms, flip angle 50°, field of view 48 × 48, 256 × 256 matrix and125 kHz bandwidth.

### CMR image analysis

**Fig. 1:**
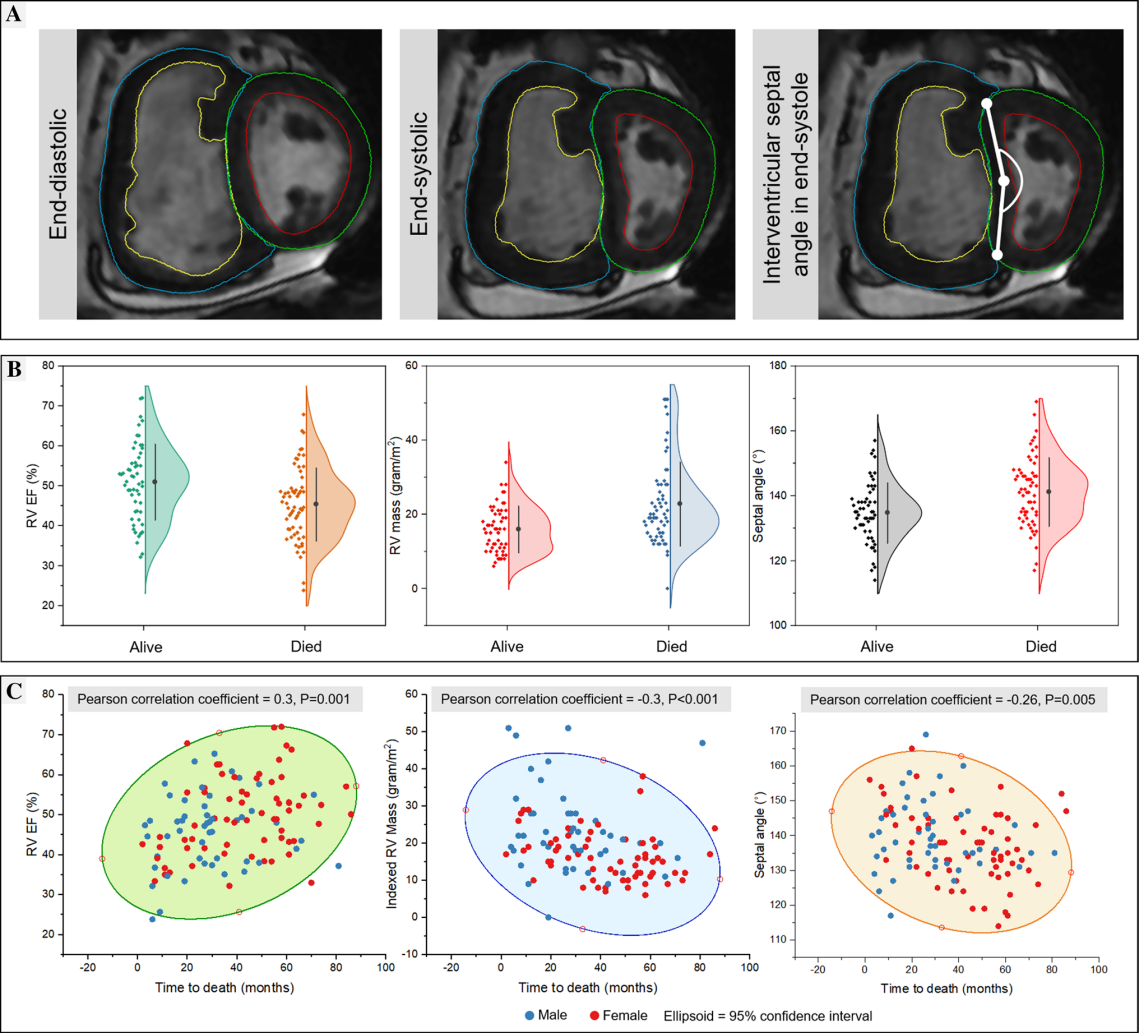
Panel **a** Illustration of study case. There is RV hypertrophy with reduced RV function (RV EF 40%) and the septal angle is 190°. Panel **b** Mean plots for RV EF, indexed mass and septal angle. Panel **c** Scatter plots for the three variables against time-to-death

**Fig. 2:**
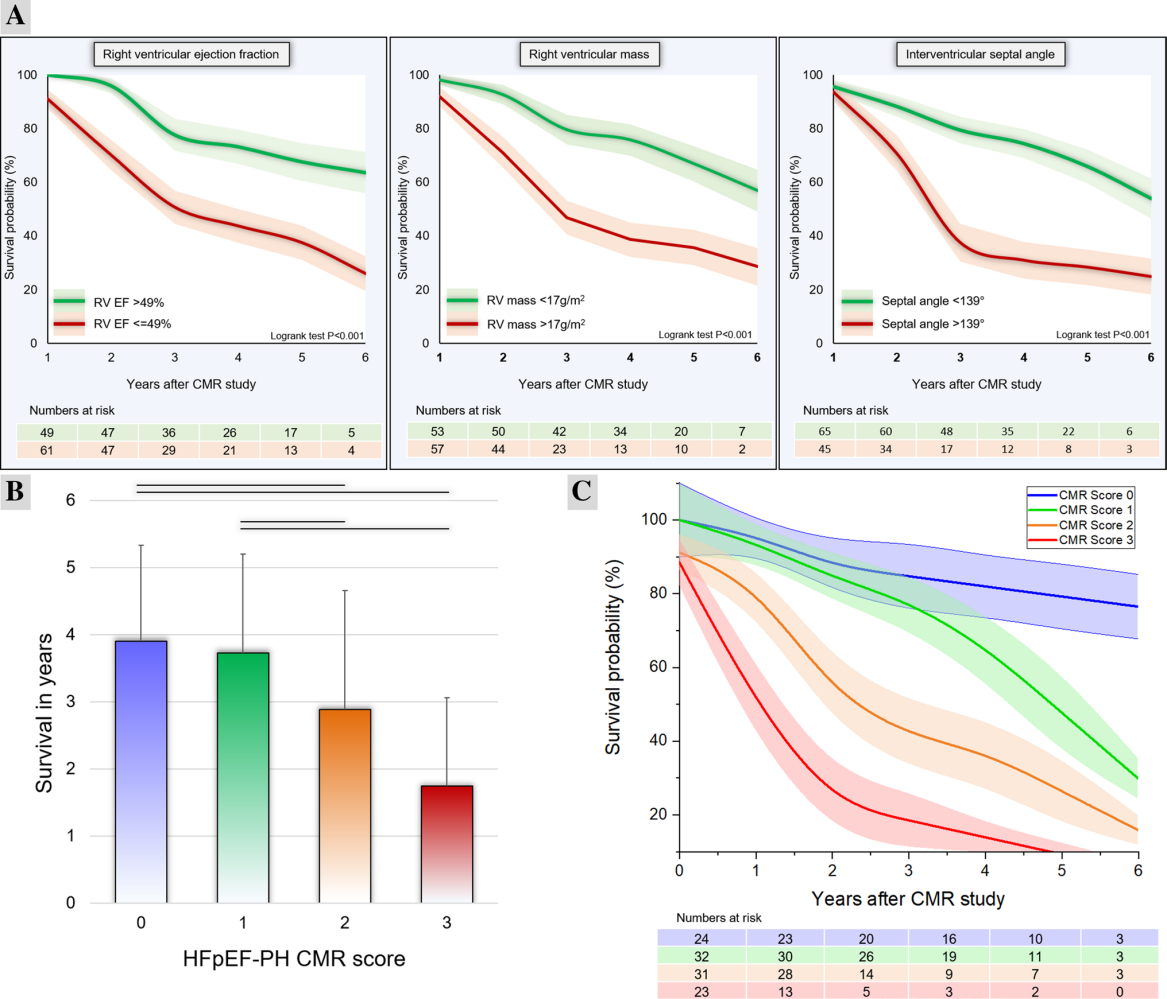
**Panel a**Kaplan-Meier survival curves. Panel **b** CMR score model predicts survival in pulmonary hypertension-HFpEF. Panel **c** Kaplan–Meier survival curve results for the HFpEF-PH CMR score

CMR images were manually analysed on GE Advantage Workstation ReportCard software, by an experienced radiographer (DC). All segmentation was done manually. Ventricular volumetric assessment was performed as per guidelines [18] (S-Fig. 1–2). All volume parameters were indexed to body surface area. Metrics included: the indexed left ventricular end-diastolic volume (LVEDVi), end-systolic volume (LVESVi), stroke volume (LVSVi), left ventricular ejection fraction (LVEF), right ventricular end-diastolic volume (RVEDVi), end-systolic volume (RVESVi), stroke volume (RVSVi) and right ventricular ejection fraction (RVEF). Left atrial volume indexed was calculated using the biplane area-length method [19]. The interventricular (IV) septal angle were measured as previously described [20]. Maximal and minimal pulmonary arterial (PA) areas were manually traced, and relative area change (RAC) was defined by the following equation: PA RAC: (maximum area–minimum area) /minimum area [21]. Reproducibility for these CMR metrics have been previously published by our group [11, 20].

### Statistics

Statistical analysis was performed using SPSS statistics 22 (IBM, Chicago). All continuous variables are presented as mean (standard deviation). Independent T-test was used to compare the clinical and CMR variables in alive/dead patients. For categorical comparisons, the Chi-square test was used. Further details on statistical analysis are in the supplementary online file. A P-value of 0.05 was considered statistically significant.

## Results

### Study population

The baseline demographics of the 116 patients with pulmonary hypertension-HFpEF who met the inclusion criteria are shown in Table 1. The average age was 73 ± 7 years and 57% of patients were female. During the mean follow-up period of 3 ± 2 years, 61 patients with pulmonary hypertension-HFpEF died (53%).

**Table 1:** Study demographics and pulmonary haemodynamics

	All	Alive	Dead	P-value*
N	116	55	61	
Age (yrs)	73 ± 7	71 ± 8	75 ± 7	0.018
Gender (Male)	50 (43%)	15 (27%)	35 (57%)	0.001
BMI (kg/m^2^)	31 ± 6	31 ± 7	30 ± 6	0.284
Heart rate (bpm)	65 ± 12	65 ± 13	65 ± 11	0.800
Diabetes mellitus	30 (26%)	13 (24%)	17 (28%)	0.607
Hypercholesterolaemia	24 (21%)	11 (20%)	13 (21%)	0.863
Hypertension	78 (67%)	41 (75%)	37 (61%)	0.056
IHD	20 (17%)	9 (16%)	11 (18%)	0.814
Atrial fibrillation	82 (71%)	32 (58%)	50 (82%)	0.009
Stroke	8 (7%)	3 (5%)	5 (8%)	0.565
Invasive haemodynamics				
Mean RA (mmHg)	15 ± 5	14 ± 5	17 ± 5	0.004
Mean PAP (mmHg)	42 ± 10	39 ± 9	45 ± 10	0.002
PCWP (mmHg)	23 ± 5	23 ± 5	22 ± 5	0.327
DPG (mmHg)	1 ± 7	-1 ± 6	3 ± 7	< 0.001
TPG (mmHg)	19 ± 9	16 ± 7	22 ± 9	< 0.001
Cardiac index (L/min/m^2^)	2.8 ± 1	2.9 ± 1.1	2.7 ± 0.8	0.432
PVR (Wood unit)	4.2 ± 2.95	3.16 ± 1.7	5 ± 3.5	< 0.001
O2 saturation (arterial)	95 ± 4	95 ± 3	94 ± 4	0.071
O2 saturation (venous)	65 ± 9	68 ± 8	63 ± 10	0.005

Categorical comparisons done by Chi-square test

*BMI* Body mass index, *IHD* Ischaemic heart disease, *RA* right atrial, *PAP* pulmonary artery pressure, *PCWP* pulmonary capillary wedge pressure, *DPG* diastolic pulmonary gradient, *TPG* transpulmonary pressure gradient, *PVR* pulmonary vascular resistance

^*^P-value is for T-Test comparison between Alive and Dead cohorts

### Left heart volumetric assessment

Left atrial volume index was significantly higher in the patients who were dead at the census (80.3 ± 31.2 ml/m^2^ vs 66.5 ± 19.1 ml/m^2^, P = 0.005), whereas, there were no significant differences in LV volumes (LVEDVi, LVESVi), LV systolic function assessed by LVEF, LV stroke volume index or LV mass (indexed) (Supplementary Table 1).

### Right heart volumetric assessment

Right ventricular volumetric parameters including RVESVi, RV mass and interventricular septal angle were significantly higher (P < 0.005) and RVEF significantly lower (P = 0.002) in patients with pulmonary hypertension-HFpEF who were dead compared to those alive at the census date (Fig. 1A).

### Pulmonary artery area

The PA diastolic area was significantly increased (P = 0.003). PA systolic area was increased (P = 0.016) and PA relative area change reduced (P = 0.027) in patients who were dead compared to those alive at the census data.

At the univariate analysis of demographic and CMR variables, 11 were associated with mortality (Supplementary Table 2). In a forward selection, multivariate cox regression model, only three parameters demonstrated independent association to all-cause mortality in this patient population. These included the CMR parameters–RVEF (HR 0.64, 95% CI 0.47 to 0.87, P < 0.001), indexed RV mass (HR 1.46, 95% CI 1.18 to 1.8, P < 0.001) and IV septal angle (HR 1.48, 95% CI 1.12 to 1.94, P < 0.001) (Fig. 1).

Pulmonary hypertension-HFpEF with RVEF less than or equal to 49% had a worse survival at 1-year (70% vs 96%), 3-years (44% vs 73%) and 5-years (26% vs 64%), than patients with a RVEF > 49%, respectively (P = 0.0001) (Fig. 2). In patients with an indexed RV mass > 17 g/m^2^ patients had a worse survival at 1-year (71% vs. 93%), 3-years (39% vs. 76%) and at 5-years (30% vs. 60%) than patients with indexed RV mass <  = 17 g/m^2^ (P = 0.0002). Patients with an inter-ventricular septal angle > 139° had a worse survival at 1-year (71% vs. 90%), 3-years (31% vs. 74%) and at 5-years (25% vs. 54%) than patients with an inter-ventricular septal angle <  = 139° (P = 0.0001). Receiver operating characteristic analysis are detailed in Table 2 and the supplementary document.

**Table 2:** C-statistics

	AUC	95% CI	P	Criterion^b^	Sensitivity (95% CI)	Specificity (95% CI)
RV EF (%)	0.67^a^	0.57 to 0.75	< 0.01	≤ 49	75 (63–85.5)	58 (44–71)
Indexed RV mass (grams/m^2^)	0.70	0.61 to 0.78	< 0.01	> 17	67 (54–79)	62 (48–75)
IV septal angle (˚)	0.67^a^	0.58 to 0.76	< 0.01	> 139	57 (44–70)	76 (63–87)
CMR model	0.76	0.67 to 0.83	< 0.01	> 1	72 (59–83)	71 (57–82)

*AUC* area under the curve, *CI* confidence interval, *RV EF* Right ventricular ejection fraction, *IV* intra-ventricular, *CMR* cardiac magnetic resonance

^a^Significantly different to CMR model AUC

^b^Youden index derived

A score was developed defined by thresholds from receiver operator characteristic (ROC) analysis. The minimum score was set at 0 which meant all the three variables were not above their threshold. The maximum score was 3 which meant all the three variables were above their threshold. The CMR score model demonstrated a higher area under the curve than the three variables individually at 0.76, 95% CI 0.67 to 0.83, P < 0.001. The overall sensitivity, specificity and accuracy of a pulmonary hypertension-HFpEF CMR score > 2 for predicting all-cause mortality were 72% (95% CI 59.2–82.9), 71% (95% CI 57.1–82.4) and 71.5% (95% CI 62.4–79.5) respectively (Fig. 2).

## Discussion

In this study we have shown that a CMR based volumetric and functional assessment of the right ventricle can risk stratify patients with pulmonary hypertension-HFpEF. In addition, we have presented a simple CMR scoring model incorporating independent predictors of outcome RV EF, indexed RV mass and IV septal angle. Our study highlights the potential value of CMR in the prognostic assessment of patients with pulmonary hypertension-HFpEF.

Echocardiography is a first-line diagnostic imaging test for patients presenting with symptoms and signs of HF [22]. Several studies have demonstrated that echocardiographic measures reflecting RV function predict prognosis in pulmonary hypertension-HFpEF [2, 23–25]. It is worth noting that the majority of echocardiography-based studies have demonstrated that it is mainly RV function and remodelling that are associated with a poor prognosis in pulmonary hypertension-HFpEF. In a study which recruited 419 patients with HFpEF, Burke et al. demonstrated that LV compliance and also RV hypertrophy (hazard ratio = 1.37; P < 0.001), were most predictive of worse outcomes [24]. Similar to our study, neither LV function nor any other LV volumetric parameter was independently associated with cardiovascular outcomes. In another large study of 562 patients which assessed RV function in a semiquantitative way, Mohammed et al. demonstrated that the presence of impaired RV function was associated with higher all-cause mortality (hazard ratio = 1.35; P = 0.03) [23]. It is worth noting that even though echocardiography is a good screening imaging modality, evaluation of right heart remains challenging and limited. Moreover, almost 10–15% patients can have non-diagnostic studies due to poor acoustic windows. The role of CMR is not only to clarify the diagnosis, but also monitor disease process longitudinally. CMR uniquely allows one to quantify RV mass precisely, which cannot be done by echocardiography. Hence, CMR is the reference standard for the evaluation of right cardiac volumes and function [26].

It is established that RV dysfunction on CMR predicts outcomes in pulmonary arterial hypertension [27]. The findings of this study are broadly consistent with Aschauer et al. who also demonstrated that right ventricular systolic dysfunction by CMR is independently associated with mortality in HFpEF [28]. However, the main differences between their work and this study are that they recruited generic HFpEF patients where as in this study we only recruited patients who had developed PH-HFpEF. Hence, it is plausible to conclude that this study has recruited more severe cases of HFpEF, who have subsequently developed PH. In addition, this study has evaluated RV functional and anatomy comprehensively including septal angle which demonstrated independent prognostic role in patients with PH-HFpEF.

In this study, we have also developed a simplified scoring tool based on three CMR metrics which may aid risk stratification of patients with pulmonary hypertension-HFpEF. The CMR score is simple and easy to integrate in routine practice. Patients with CMR score > 2, could be offered more regular clinical monitoring as evidence suggests that a strategy to reduce the pulmonary artery pressure primarily by diuretic therapy can improve outcomes in these patients [29, 30]. Hence, a non-invasive CMR model which can appropriately risk stratify patients by assessing right ventricular function and the severity of pre-capillary pulmonary hypertension may be of value in clinical trials of new therapies or treatment approaches including closer monitoring and optimisation of heart failure in high-risk patients.

## Limitations

Patients were required to have a cardiac catheterisation to identify patients with HFpEF with pulmonary hypertension. The results of our study, therefore, apply to a selected cohort, however, this has the advantage of using reference standard haemodynamic measures to define pulmonary hypertension-HFpEF. HFpEF patients with a detectable scar, fibrosis on T1-mapping, myocardial ischaemia or right ventricular impairment appear to have a worse prognosis [31, 32]. In our study, we only evaluated CMR cine related parameters. Further studies are needed to evaluate prognostic role of multi-parametric mapping in pulmonary hypertension-HFpEF. However, we feel the current CMR model has value in patients who either have contraindication for gadolinium contrast agent or are due to have a shorter CMR scan for monitoring of function. Finally, this study did not record echocardiography data to evaluate incremental role of CMR.

## Conclusion

In this study, we observed that CMR can risk stratify pulmonary hypertension-HFpEF patients and predict all-cause mortality. When patients develop pulmonary hypertension associated with HFpEF, it is primarily right heart function and imaging features of pre-capillary pulmonary hypertension which predict mortality.

## Supplementary Information

Below is the link to the electronic supplementary material.

Supplementary file1 (DOCX 3511 KB)
