# Supplementary material for: Cardiovascular magnetic resonance predicts all-cause mortality in pulmonary hypertension associated with heart failure with preserved ejection fraction
Source: Int J Cardiovasc Imaging. 2021 May 12;37(10):3019–25. doi: 10.1007/s10554-021-02279-z (PMC8494694; doi:10.1007/s10554-021-02279-z)
Supplement: Supplementary file 1 — Supplementary file1 (DOCX 3511 KB) [file 10554_2021_2279_MOESM1_ESM.docx]

**Supplementary document**

**Statistics**

The interval from CMR until all cause of death or census was regarded as the follow-up period. The census was performed on 22nd May 2017. Log-log plots were inspected to ensure linearity with outcome data. Univariate and multivariate Cox proportional hazards regression was performed `with each variable standardized as the z score for the population studied, in order to allow for comparison between variables. Univariate and multivariate Cox proportional hazards regression was performed. Multivariate analysis was performed in a forward direction, for all variables with a statistically significant association with mortality in univariate analysis. C-statistics were used to perform receiver operator characteristics (ROC) curve analysis and Youden criterion was used to determine thresholds for the variables which demonstrated independent association to mortality. Kaplan-Meier plots were generated using the above-derived thresholds. Log-rank χ2 was calculated for the Kaplan Meier data. A p-value of 0.05 was considered statistically significant.

**Receiver operating characteristic**

Of the three variables independently associated with all-cause mortality, the area under the curve for predicting mortality for RV EF was 0.67, indexed RV mass 0.7 and IV septal angle 0.67 (S Fig 3). The Youden index derived threshold for RV EF was <=49%, indexed RV mass >17gram/m^2^ and IV septal angle >139 ˚. Kaplan-Meier survival curves are presented in Figure 4. Pulmonary hypertension-HFpEF with RV EF less than or equal to 49% had a worse survival at 1-year (70% vs 96%), 3-years (44% vs 73%) and 5-years (26% vs 64%), than patients with a RV EF >49%, respectively (p=0.0001). In patients with an indexed RV mass >17gram/m^2^ patients had a worse survival at 1-year (71% vs 93%), 3-years (39% vs 76%) and at 5-years (30% vs 60%) than patients with indexed RV mass <=17gram/m2 (p=0.0002). Patients with an inter-ventricular septal angle >139 ̊ had a worse survival at 1-year (71% vs 90%), 3-years (31% vs 74%) and at 5-years (25% vs 54%) than patients with an inter-ventricular septal angle <=139 ̊ (p=0.0001).

**Supplementary Table 1.** CMR assessment in the study population.

|  | **All** | **Alive (n=55)** | **Dead (n=61)** | **P-value** |
| --- | --- | --- | --- | --- |
| **Left ventricular volume and function** | | | | |
| LV EDVi (ml/m^2^) | 71.1±20.4 | 72.9±19.9 | 69.5±20.9 | 0.371 |
| LV ESVi (ml/m^2^) | 24±11.1 | 24.2±10.8 | 23.8±11.4 | 0.860 |
| LV EF (%) | 67±8.1 | 67.3±8.1 | 66.7±8.2 | 0.694 |
| LV SVi (ml/m^2^) | 47.1±12.2 | 48.8±12.3 | 45.6±12 | 0.168 |
| LV mass (gram/m^2^) | 58.5±18.2 | 56.7±17.3 | 60.3±19.1 | 0.306 |
| LA volume indexed (ml/m^2^) | 73.8±27 | 66.5±19.1 | 80.3±31.2 | 0.005 |
| **Right ventricular volume and function** | | | | |
| RV EDVi (ml/m^2^) | 93.4±34.2 | 85.3±32.6 | 100.7±34.3 | 0.014 |
| RV ESVi (ml/m^2^) | 48.8±20.3 | 42.3±19.3 | 54.7±19.5 | <0.001 |
| RV SVi (ml/m^2^) | 44.6±18.1 | 43±17 | 46±19.1 | 0.376 |
| RV EF (%) | 50±10 | 51±10 | 45±9 | 0.002 |
| RV mass (gram/m^2^) | 19.6±9.8 | 16±6.3 | 22.9±11.3 | <0.001 |
| **Other metrics** | | | | |
| IV septal angle (˚) | 138.2±10.4 | 134.8±9.2 | 141.2±10.6 | <0.001 |
| PA systolic area (cm^2^) | 925.8±254 | 866.3±230.4 | 979.5±264 | 0.016 |
| PA diastolic area(cm^2^) | 827.8±222.1 | 764.6±203.1 | 884.8±224.6 | 0.003 |
| PA relative area change (%) | 12±7.3 | 13.6±7.2 | 10.6±7.2 | 0.027 |

**Supplementary Table 3.** Cox regression for all the clinical and imaging variables associated with mortality.

|  | Uni-variate | | | | Multi-variate | | | |
| --- | --- | --- | --- | --- | --- | --- | --- | --- |
|  | **HR** | **95% CI** | **Wald** | **P** | **HR** | **95% CI** | **Wald** | **P** |
| Age (yrs) | 1.33 | 0.99 to 1.78 | 3.6 | 0.057 |  |  |  |  |
| Gender (Male) | 2.74 | 1.63 to 4.61 | 14.5 | <0.001 |  |  |  |  |
| Atrial fibrillation | 2.11 | 1.11 to 3.98 | 5.3 | 0.021 |  |  |  |  |
| LA volume (ml/m^2^)* | 1.32 | 1.07 to 1.64 | 6.7 | 0.010 |  |  |  |  |
| RV EDV (ml/m^2^) | 1.30 | 1.03 to 1.64 | 4.8 | 0.029 |  |  |  |  |
| RV SV (ml/m^2^)* | 1.47 | 1.17 to 1.85 | 11.1 | <0.001 |  |  |  |  |
| RV EF (%) | 0.58 | 0.44 to 0.78 | 13.6 | <0.001 | 0.64 | 0.47 to 0.87 | 8.0 | <0.001 |
| RV mass (gram/m^2^)* | 1.55 | 1.26 to 1.9 | 17.4 | <0.001 | 1.46 | 1.18 to 1.8 | 12.4 | <0.001 |
| IV septal angle (˚) | 1.58 | 1.23 to 2.03 | 12.9 | <0.001 | 1.48 | 1.12 to 1.94 | 7.8 | <0.001 |
| PA systolic area (cm^2^) | 1.29 | 1.02 to 1.64 | 4.6 | 0.038 |  |  |  |  |
| PA diastolic area (cm^2^) | 1.39 | 1.1 to 1.75 | 7.6 | 0.006 |  |  |  |  |
| PA relative area change (%) | 0.71 | 0.54 to 0.93 | 6.3 | 0.012 |  |  |  |  |

*indexed for body surface area.

**S. Figure 1.** Demonstrates right ventricular and left ventricular segmentation in a patient with no PH.


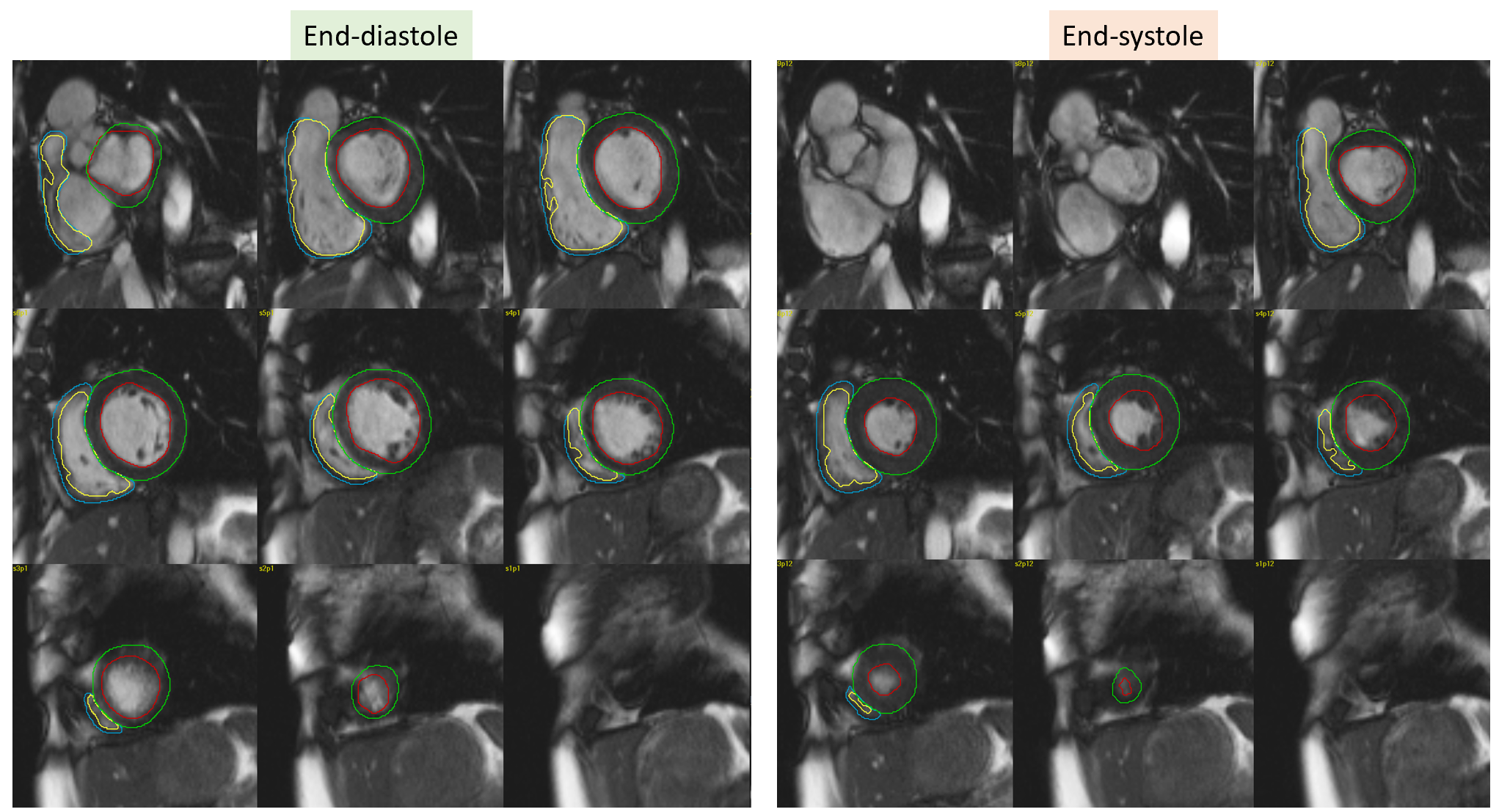


**S. Figure 2.** Demonstrates right ventricular and left ventricular segmentation in a HFpEF-PH patient.


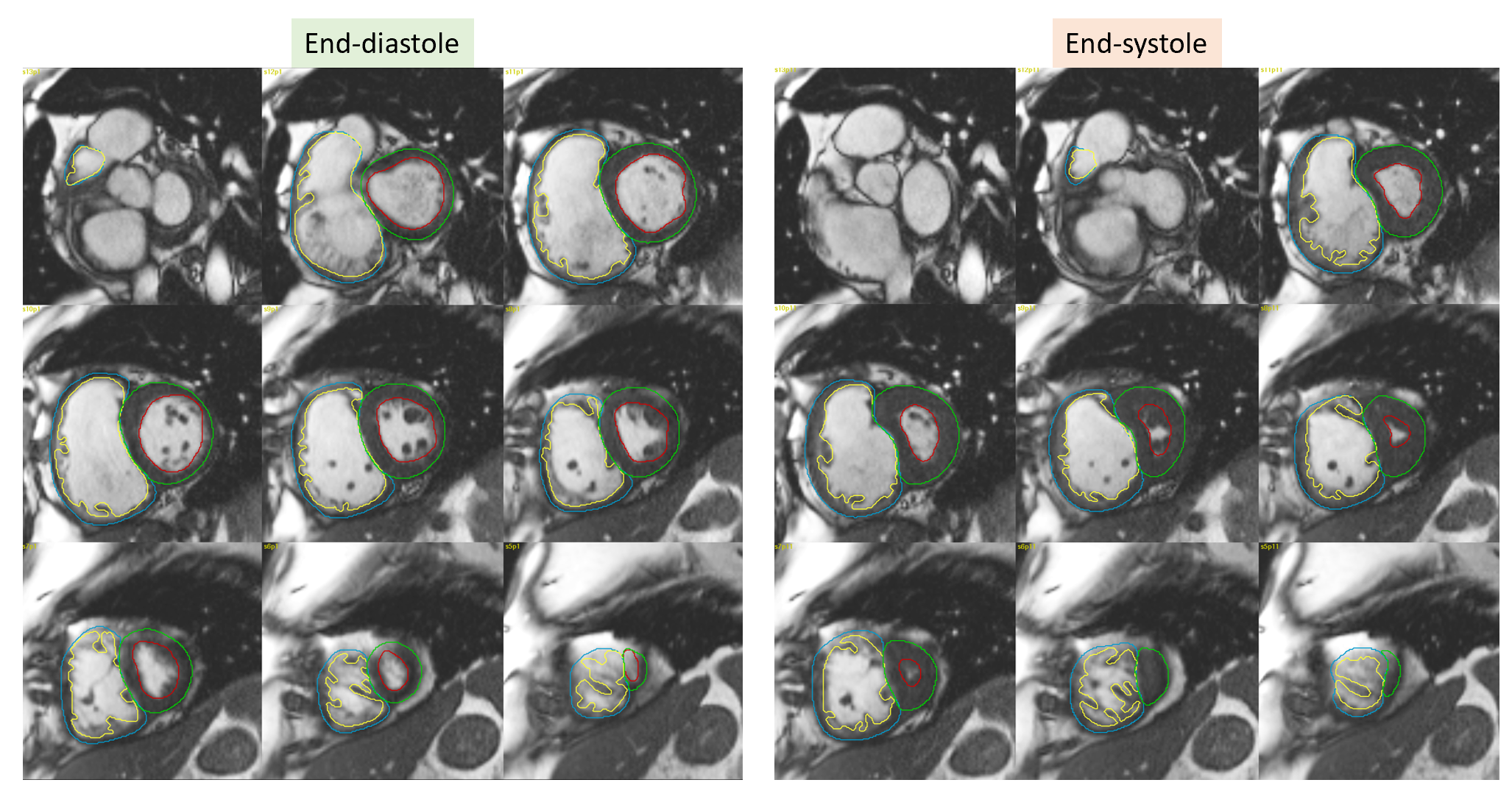


**S. Figure 3.** C-statistics - receiver operating characteristic curve (ROC) analysis results.


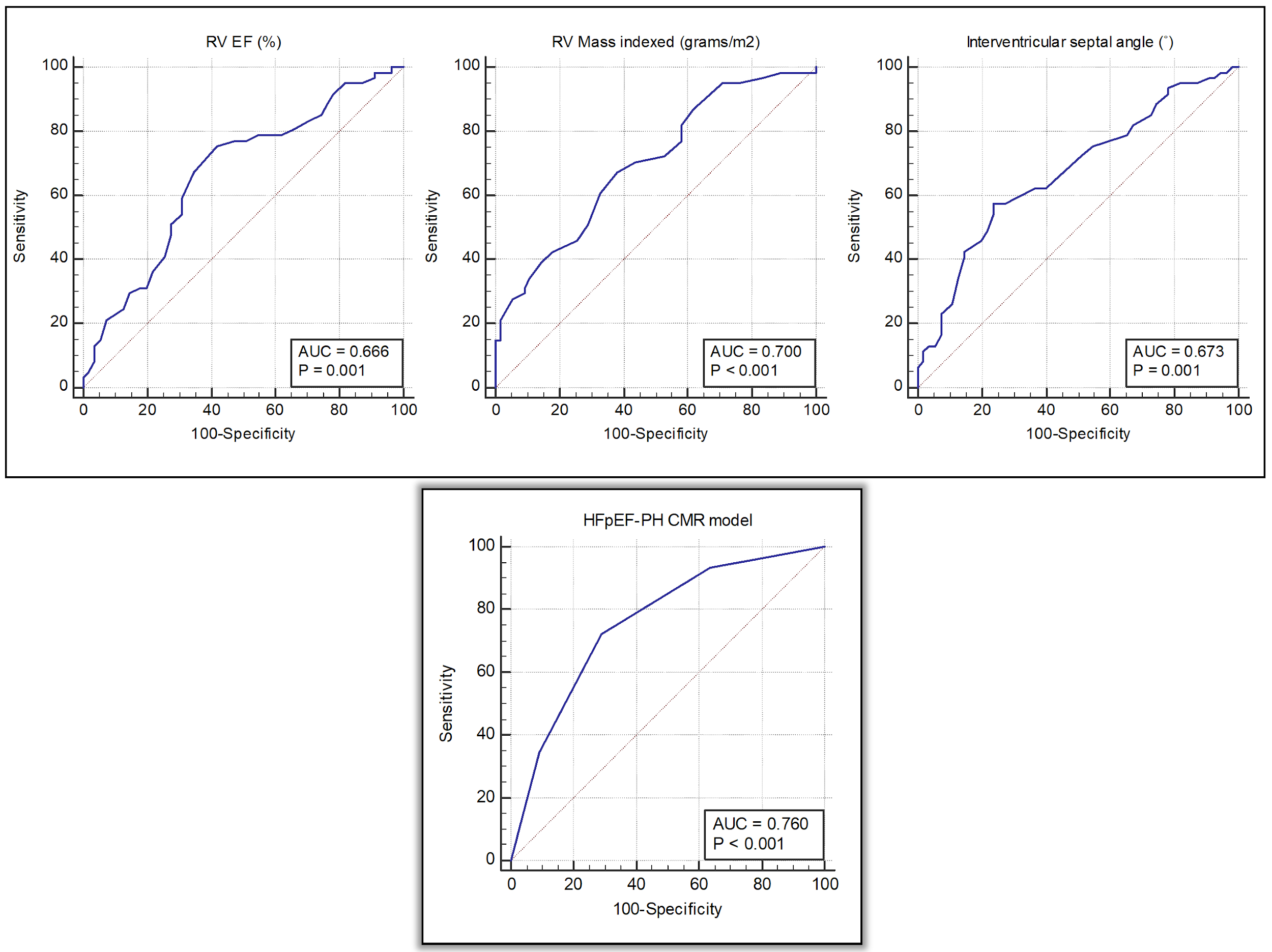


**References**

1. Kramer CM, Barkhausen J, Flamm SD, Kim RJ, Nagel E. Standardized cardiovascular magnetic resonance imaging (CMR) protocols, society for cardiovascular magnetic resonance: board of trustees task force on standardized protocols. J Cardiovasc Magn Reson Off J Soc Cardiovasc Magn Reson. 2008 Jan 7;10(1):35.

2. Khan MA, Yang EY, Zhan Y, Judd RM, Chan W, Nabi F, et al. Association of left atrial volume index and all-cause mortality in patients referred for routine cardiovascular magnetic resonance: a multicenter study. J Cardiovasc Magn Reson Off J Soc Cardiovasc Magn Reson. 2019 07;21(1):4.

3. Johns CS, Kiely DG, Rajaram S, Hill C, Thomas S, Karunasaagarar K, et al. Diagnosis of Pulmonary Hypertension with Cardiac MRI: Derivation and Validation of Regression Models. Radiology. 2018 Oct 23;180603.

4. Swift AJ, Rajaram S, Condliffe R, Capener D, Hurdman J, Elliot C, et al. Pulmonary artery relative area change detects mild elevations in pulmonary vascular resistance and predicts adverse outcome in pulmonary hypertension. Invest Radiol. 2012 Oct;47(10):571–7.

5. Swift AJ, Capener D, Johns C, Hamilton N, Rothman A, Elliot C, et al. Magnetic Resonance Imaging in the Prognostic Evaluation of Patients with Pulmonary Arterial Hypertension. Am J Respir Crit Care Med. 2017 15;196(2):228–39.

6. Johns CS, Wild JM, Rajaram S, Tubman E, Capener D, Elliot C, et al. Identifying At-Risk Patients with Combined Pre- and Postcapillary Pulmonary Hypertension Using Interventricular Septal Angle at Cardiac MRI. Radiology. 2018;289(1):61–8.
